# Identification of genes involved in shea butter biosynthesis from *Vitellaria paradoxa* fruits through transcriptomics and functional heterologous expression

**DOI:** 10.1007/s00253-019-09720-3

**Published:** 2019-03-26

**Authors:** Yongjun Wei, Boyang Ji, Verena Siewers, Deyang Xu, Barbara Ann Halkier, Jens Nielsen

**Affiliations:** 1 School of Pharmaceutical Sciences, Key Laboratory of State Ministry of Education, Key Laboratory of Henan province for Drug Quality Control and Evaluation, Collaborative Innovation Center of New Drug Research and Safety Evaluation, Zhengzhou University, 100 Kexue Avenue, Zhengzhou, 450001 Henan China; 2 Department of Biology and Biological Engineering, Chalmers University of Technology, SE-41296 Gothenburg, Sweden; 3 Novo Nordisk Foundation Center for Biosustainability, Chalmers University of Technology, SE-41296 Gothenburg, Sweden; 4 DynaMo Center, Department of Plant and Environmental Sciences, University of Copenhagen, Thorvaldsensvej 40, 1871 Frederiksberg C, Denmark; 5 Novo Nordisk Foundation Center for Biosustainability, Technical University of Denmark, Kgs., DK-2800 Lyngby, Denmark

**Keywords:** Shea butter, Shea transcriptomic, Yeast cell factories, TAG biosynthetic pathway, Synthetic biology

## Abstract

Shea tree (*Vitellaria paradoxa*) is one economically important plant species that mainly distributes in West Africa. Shea butter extracted from shea fruit kernels can be used as valuable products in the food and cosmetic industries. The most valuable composition in shea butter was one kind of triacylglycerol (TAG), 1,3-distearoyl-2-oleoyl-glycerol (SOS, C18:0–C18:1–C18:0). However, shea butter production is limited and little is known about the genetic information of shea tree. In this study, we tried to reveal genetic information of shea tree and identified shea TAG biosynthetic genes for future shea butter production in yeast cell factories. First, we measured lipid content, lipid composition, and TAG composition of seven shea fruits at different ripe stages. Then, we performed transcriptome analysis on two shea fruits containing obviously different levels of SOS and revealed a list of TAG biosynthetic genes potentially involved in TAG biosynthesis. In total, 4 glycerol-3-phosphate acyltransferase (GPAT) genes, 8 lysophospholipid acyltransferase (LPAT) genes, and 11 diacylglycerol acyltransferase (DGAT) genes in TAG biosynthetic pathway were predicted from the assembled transcriptome and 14 of them were cloned from shea fruit cDNA. Furthermore, the heterologous expression of these 14 potential GPAT, LPAT, and DGAT genes in *Saccharomyces cerevisiae* changed yeast fatty acid and lipid profiles, suggesting that they functioned in *S. cerevisiae*. Moreover, two shea DGAT genes, *VpDGAT1* and *VpDGAT7*, were identified as functional DGATs in shea tree, showing they might be useful for shea butter (SOS) production in yeast cell factories.

**Electronic supplementary material:**

The online version of this article (10.1007/s00253-019-09720-3) contains supplementary material, which is available to authorized users.

## Introduction

Shea butter is a valuable product in the cosmetic industry; it also can be used as a cocoa butter substitute (CBS) in the chocolate industry (Jahurul et al. 2013). Shea butter was extracted from the kernels of shea tree (*Vitellaria paradoxa*) fruits (Davrieux et al. 2010; Jahurul et al. 2013). Usually, it represents 40–55% of the dry weight of the ripe shea tree fruits (Davrieux et al. 2010). Shea butter contains high-levels of triacylglycerols (TAGs) with C18 fatty acids, i.e., 1,3-distearoyl-2-oleoyl-glycerol (SOS, C18:0–C18:1–C18:0, ~ 42%), 1-stearoyl-2,3-dioleoyl-glycerol (SOO, C18:0–C18:1–C18:1, ~ 26%), and trioleoyl glycerol (OOO, C18:1–C18:1–C18:1, ~ 11%) (Di Vincenzo et al. 2005; Honfo et al. 2014). Cocoa butter (CB) is mainly composed of three different kinds of TAGs, 1,3-dipalmitoyl-2-oleoyl-glycerol (POP, C16:0–C18:1–C16:0, 17.5–22.6%), 1-palmitoyl-3-stearoyl-2-oleoyl-glycerol (POS, C16:0–C18:1–C18:0, 35.8–41.4%), and SOS (22.8–31.3%) (Chaiseri and Dimick 1989; Lipp and Anklam 1998). Among the CB or CBS TAGs, SOS is the most valuable component, for that the addition of a small amount of SOS or SOS-rich TAGs to cocoa butter or cocoa butter–derived products would lead to an increased melting point and decreased tempering time of the products (Jahurul et al. 2013). However, CB and CBS derived from plants and their supply are limited. Therefore, developing other strategies, such as microbial cell factories, for CB (SOS) production is of interest (Clough et al. 2009; Wei et al. 2018).

Previous genomic analyses revealed several cocoa genes of glycerol-3-phosphate acyltransferase (GPAT), lysophospholipid acyltransferase (LPAT), and diacylglycerol acyltransferase (DGAT) participating in TAG biosynthetic pathway (Argout et al. 2011; Chapman and Ohlrogge 2012; Motamayor et al. 2013; Napier et al. 2014). Further expressing some of them in *Saccharomyces cerevisiae* increased yeast lipid or TAG production (Wei et al. 2018; Wei et al. 2017a). As shea butter contains higher SOS than cocoa butter (Chaiseri and Dimick 1989; Di Vincenzo et al. 2005; Jahurul et al. 2013), shea tree might harbor more efficient TAG biosynthetic genes than cocoa corresponding genes for SOS production. In this situation, functional characterization of lipid biosynthetic genes in shea fruits might provide insights into shea butter biosynthesis and thus candidate genes for CB (SOS) production in plants or engineered microbes (Wei et al. 2018; Wei et al. 2017a). However, little is known about the genetic information involved in lipid metabolism of shea tree (Abdulai et al. 2017; Allal et al. 2008; Fontaine et al. 2004; Kelly et al. 2004; Sanou et al. 2005).

To characterize potential TAG (SOS)-producing genes from shea tree, we compared lipid content and composition of seven shea fruits and performed further transcriptomic analyses of two shea fruits. By cloning and characterizing TAG biosynthetic genes of GPAT, LPAT, and DGAT in shea fruit, we identified several functional TAG biosynthetic genes. Among them, two DGAT genes were believed to be used for TAG production in shea tree.

## Materials and methods

### Plant materials

The shea tree for sampling was located in Africa. Seven different shea fruits named T1 to T7 were harvested randomly from the same tree with intervals of 5–20 days between April 2015 and June 2015 (Table S1). The fresh shea fruit samples were immediately covered with aluminum foil after harvest and stored in plastic zip-lock bags at − 20 °C, and they were transported to the laboratory in Göteborg while being kept at − 20 °C in June 2015. Though leaves were also sampled from the same tree, they were dehydrated and it was not possible to extract intact RNA from them.

### Lipid extraction

The weight of each fruit was determined with a balance. The shea fruit pulp, shell, and kernel (the origin of shea butter) were separated with a sterilized scalpel. The kernel of each shea fruit was ground to very fine powder in liquid N_2_ using mortar and pestle and the powder weight was determined. Then, 6-mL methanol/chloroform 1:1 (*v*/*v*) solution per g of kernel was added and the samples were incubated for 10 min at 1500 rpm using a DVX-2500 multi-tube vortexer (VWR). After centrifugation at 6500×*g* for 10 min, the lower phase (chloroform phase) was collected into a new 50-mL falcon tube. In order to extract all the lipids, another equal volume of chloroform was added to the upper phase, mixed using a DVX-2500 multi-tube vortexer, and incubated for 10 min at 1500 rpm. After centrifugation at 6500×*g* for 10 min, the lower phase was collected and combined with the previously obtained lower phase. Finally, an equal volume of 0.1% NaCl was added to the combined lower phases (chloroform phase), vortexed, and centrifuged at 6500×*g* for 10 min to collect the lower phase. The collected lower phase liquid was dried in glass tubes with a MiVac concentrator (Genevac) at 50 °C until the weight of each sample did not change. Fatty acid methyl ester (FAME), lipid, and TAG profiles were analyzed as described before (Wei et al. 2017a; Wei et al. 2017b).

For yeast strains, 100 mL of shake flasks containing 20 mL minimal medium was used to carry out the shake flask fermentations for lipid and fatty acid analyses, and the details were described before (Wei et al. 2017a). Some strains were cultivated in 5-L shake flasks containing 1 L NLM medium to obtain sufficient lipids for TAG analyses (Wei et al. 2017b). The fatty acid methyl ester (FAME), lipid, and TAG profiles of each sample were analyzed as described before (Khoomrung et al. 2012; Khoomrung et al. 2013; Wei et al. 2018; Wei et al. 2017a; Wei et al. 2017b).

### RNA preparation and sequencing

The total RNA of two fruits T3 and T6 was extracted from the previously prepared 100 mg fine powder (see above). A 0.5-mL cold (4 °C) PureLink plant RNA reagent (Life Technologies) was added to each 100 mg plant sample. The suspension was briefly mixed by vortexing until the shea sample was thoroughly resuspended and incubated for 5 min at room temperature. The solution was clarified by centrifuging at 12,000×*g* in a microcentrifuge for 2 min at room temperature. Finally, the lysate was transferred to a QIAshredder spin column placed in a 2-mL collection tube and the instructions of the RNeasy plant mini kit (Qiagen) were followed to extract total RNA. RNA quality was checked with a 2100 Bioanalyzer (Agilent) and sent to GATC Biotech (Germany) for further Illumina 2 × 125-bp paired-end sequencing. Moreover, little amount of extracted shea RNA was converted into cDNA using Qiagen QuantiTect Reverse Transcription Kit.

### RNA-seq data analyses

The Fastq format raw reads were processed through our Perl scripts. In this step, low-quality reads were discarded and adaptor sequences were trimmed. All the downstream analyses were based on clean data with high quality. Each side of the cleaned raw reads was pooled together and assembled into contigs using Trinity (Haas et al. 2013). The min_kmer_cov of the assembly parameter was 2 and all other parameters were set as default values. Genes in each assembly contig were annotated with UniProt (The UniProt 2017). The TPM (transcripts per kilobase million) value of each gene was calculated based on each gene length and its respective total RPK (reads per kilobase) value.

### Bioinformatics analyses of shea GPAT, LPAT, and DGAT genes

Full-length protein sequences of *Arabidopsis thaliana* lipid metabolic genes were downloaded from the *Arabidopsis* acyl-lipid metabolism database (http://aralip.plantbiology.msu.edu/pathways/pathways). The *Arabidopsis* protein sequences were used to query the assembled shea transcriptomic contigs using the TBLASTN program. An *E*-value cutoff of *e*^−12^ was used to identify the acyl carrier protein (ACP) gene family due to their short length (< 150 amino acids), while an *E*-value of *e*^−24^ was used to identify other genes (Argout et al. 2011). Moreover, to identify all the potential full-length GPAT, LPAT and DGAT gene sequences of shea tree, reference GPAT, LPAT and DGAT gene sequences of *Arabidopsis thaliana*, *S. cerevisiae* and cocoa tree were downloaded from the KEGG database and used to query the assembled shea contigs (Kanehisa et al. 2016). A total of 23 potential genes, including 4 GPAT genes, 8 LPAT genes, and 11 DGAT genes, were annotated and deposited in the GenBank database under the accession numbers MG280902-MG280924. Then, amino acid sequences of all the potential GPAT, LPAT, or DGAT were aligned using the MAFFT (Katoh and Standley 2013), and the multiple alignment results were used to create phylogenetic trees using the MEGA 7.0.21 software (Kumar et al. 2016). The neighbor-joining method with the Poisson correction was used to create the phylogenetic tree with bootstrap confidence values of 1000 replicates. Moreover, gaps in the alignment of GPAT, LPAT, and DGAT sequences were treated with the pair wise-deletion option.

### Strains, plasmids, and media

The *S. cerevisiae* strain IMX581 (*MAT*a *ura3-52 can1∆*::*cas9*-*natNT2 TRP1 LEU2 HIS3*) and *S. cerevisiae* strain Y29 (IMX581 sct1Δ ale1Δ lro1Δ dga1Δ) were used for shea tree gene expression in this study (Mans et al. 2015; Wei et al. 2018). All yeast strains constructed and used in this study are listed in Table S2, and primers used to construct the yeast strains are listed in Table S3.

### Expression of shea TAG biosynthetic genes in yeast

Shea genes were amplified from shea cDNA using the PrimeSTAR HS DNA polymerase (Takara) according to the manufacturer’s instruction. The obtained genes were expressed under control of the promoters of *TEF1*, *PGK1*, or *FBA1* and the terminators of *ADH1*, *GAT2*, or *CYC1*, respectively (Figure S1 and Table S2). Gibson assembly (NEB) was used to construct cocoa gene expression plasmids by ligation of the gene expression cassettes and the amplified linear backbone fragment of plasmid pRS416 (Sikorski and Hieter 1989) and was further verified by PCR and Sanger sequencing. *S. cerevisiae* was transformed with the verified plasmids and the obtained strains are listed in Tables S2 and S3.

## Results

### The lipid profiles of shea fruits

**Fig. 1:**
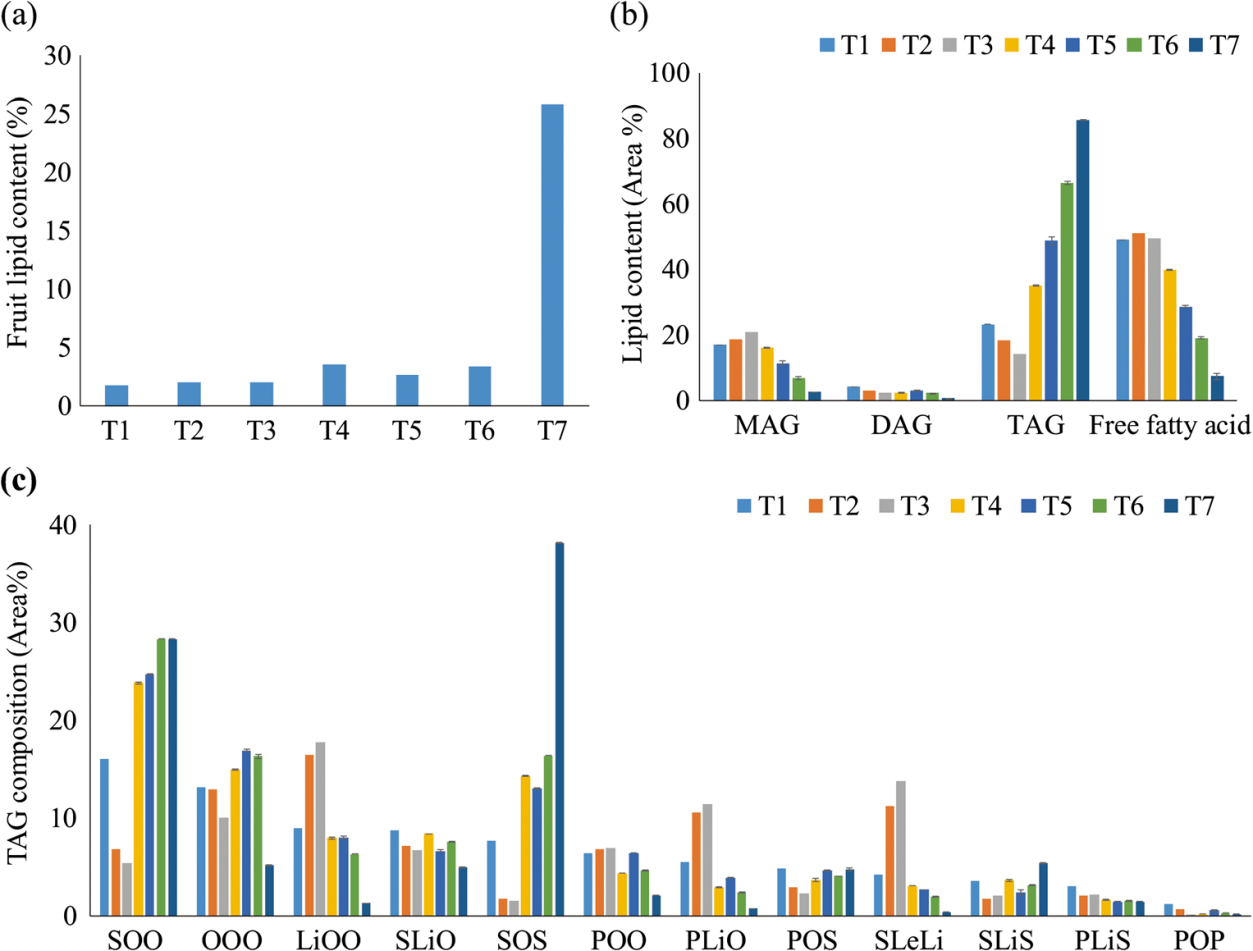
Shea fruit lipid content analyses. **a** Relative lipid contents of seven shea fruits harvested in 7 different days from one shea tree (*Vitellaria paradoxa*) were shown. % represents g lipids/g weight. **b** Lipid profiles and its relative content of seven shea fruits harvested from one Shea tree (*Vitellaria paradoxa*) were shown. MAG, monoacylglycerol; DAG, diacylglycerol; TAG, triacylglycerol. The error bars of fruits T4 to T7 represented two technical replicates, and there are not enough lipids for T1 and T3 to do the technical replicates. **c** TAG profiles (> 2% total TAGs) of seven shea fruits were shown. P, palmitic acid C16:0; S, steric acid C18:0; O, oleic acid C18:1; Li, linoelaidic acid C18:2; Le, linolenic acid C18:3. The error bars of fruit T4 to T7 represented two technical replicates, and there are not enough lipids for T1 and T3 to do the technical replicates

The lipid content, lipid composition, and TAG composition of T1 to T7 were measured (Fig. 1) (Table S1). The lipid contents of sample T1–T7 ranged from 2 to 25% (Fig. 1a). Total lipid profiles of the seven fruits (Fig. 1b) were different as did the corresponding relative TAG compositions (Fig. 1c). The relative TAG content increased from T4 to T7, while monoacylglycerol (MAG), diacylglycerol (DAG), and fatty acid proportions decreased from T3 to T7 (Fig. 1b). Concerning TAG composition, relative amounts of SOS and SOO increased from T1 to T7, while the proportion of other TAGs, such as PLiO (C16:0–C18:2–C18:1) decreased in T7 (Fig. 1c and Table S4). Moreover, relative fatty acid amounts of these seven fruits were different, such as C18:0 fatty acid in the TAGs increased from T5 to T7, while some fatty acids (including polyunsaturated fatty acids of C18:2 and C18:3) decreased from T3 to T7, showing that the fatty acid production profiles of the TAGs were different in these shea fruits (Figure S2). The SOO and SOS proportions in sample T3 were 5.4% and 1.6%, respectively, while their contents in sample T4 to T7 increased to more than 20% and more than 13%, respectively.

### Identification of shea fruit TAG biosynthetic genes from transcriptome

**Fig. 2:**
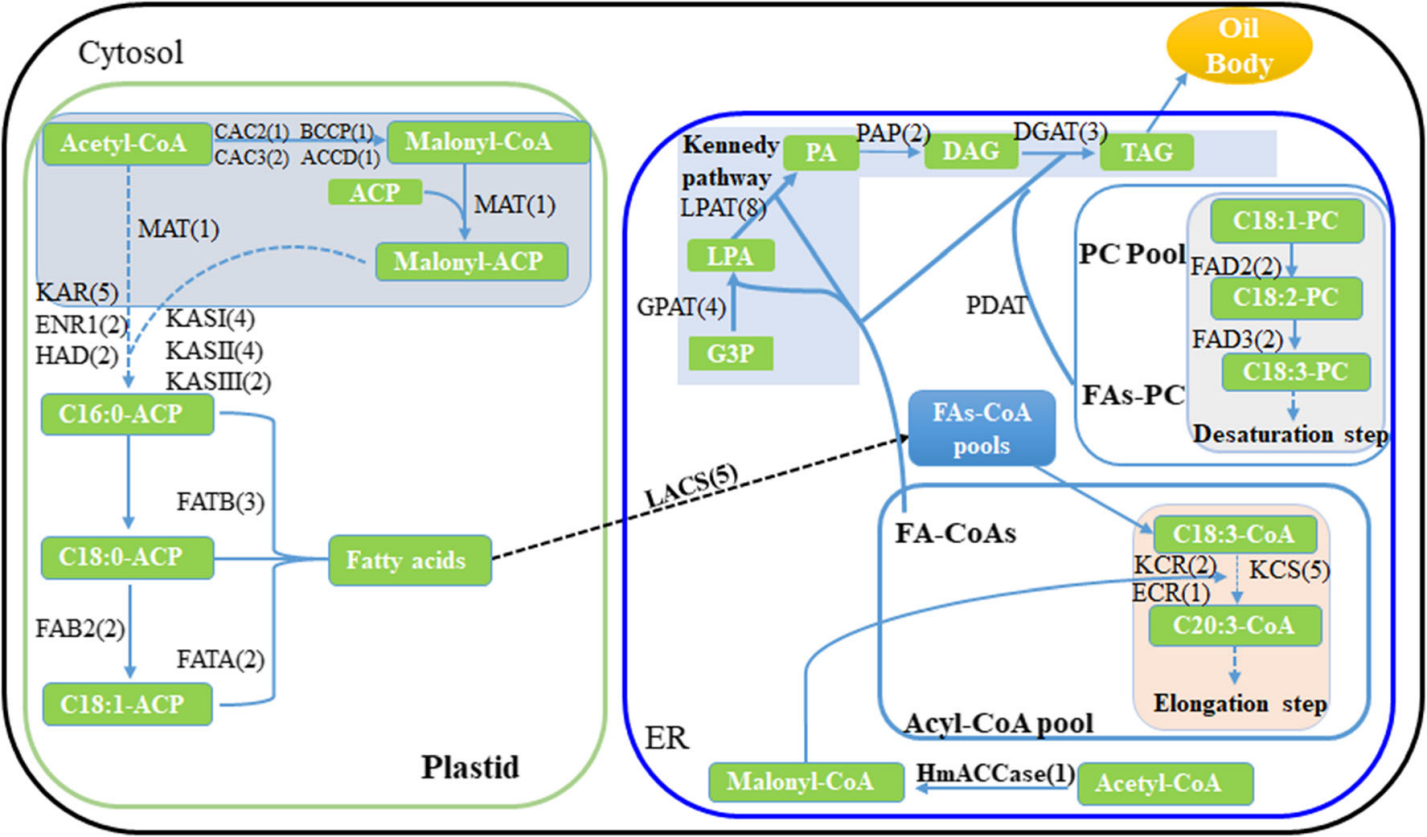
Proposed metabolic pathway for shea butter biosynthesis adapted from Argout et al. 2011 and Baud and Lepiniec 2010. The genes for shea butter biosynthesis were marked and the gene numbers were labeled after each gene. Enzymes involved in the pathway are listed based on their sequential order and their compartmentalization in plastid and ER. Predicted orthologous gene copy numbers in shea tree are indicated in parentheses beside each enzyme abbreviation: CAC2, heteromeric acetyl-CoA carboxylase BC subunit; BCCP, heteromeric acetyl-CoA carboxylase BCCP subunit; CAC3, heteromeric acetyl-CoA carboxylase alpha-CT subunit; ACCD, heteromeric acetyl-CoA carboxylase beta-CT subunit; ACP, acyl carrier protein; CoA, coenzyme A; MAT, plastidial malonyl-CoA; ACP malonyltransferase; KAS, ketoacyl-ACP synthase; KAR, plastidial ketoacyl-ACP reductase; HAD, plastidial hydroxyacyl-ACP dehydrase; ENR1, plastidial enoyl-ACP reductase; FAB2, stearoyl-ACP desaturase; FATA, acyl-ACP thioesterase; FATB, acyl-ACP thioesterase; LACS, long-chain acyl-CoA synthetase; FAD2, ER oleate desaturase; FAD3, ER linoleate desaturase; KCS, β-Ketoacyl-CoA synthase; KCR, ketoacyl-CoA reductase; ECR, enoyl-CoA reductase; HmACCase, homomeric acetyl-CoA carboxylase; LPCAT, lysophosphatidylcholine acyltransferase; GPAT, glycerol-3-phosphate acyltransferase; LPAT, lysophosphatidic acid acyltransferase; PAP, phosphatidic acid phosphatase; DGAT, acyl-CoA:diacylglycerol acyltransferase; G3P, glycerol-3-phosphate; TAG: triacylglycerol. CAC2, BCCP, CAC3, and ACCD are the four subunits of ACCase in the plastid. Dashed arrows indicate the four-step elongation cycle catalyzed by KAS, KAR, HAD, and ENR1, which is repeated multiple times during chain elongation. Orthologous gene number for each enzyme in *T. cacao* was determined as described

To identify the potential lipid metabolic genes in shea tree, deep sequencing of total RNA from two different shea fruit samples of T3 and T6 were performed. It showed that the obtained shea tree transcriptome harbored all the necessary genes for plant TAG biosynthesis. Most of the identified TAG biosynthetic gene numbers were close to the reported numbers in *A*. *thaliana* and *Theobroma cacao* (Fig. 2 and Table 1) (Argout et al. 2011; Baud and Lepiniec 2010; Motamayor et al. 2013). Moreover, the beta-ketoacyl-CoA synthase (KCS) gene number of shea tree is lower than *A*. *thaliana* and *T*. *cacao* (Table 1) (Argout et al. 2011).

**Table 1:** Comparison of genes orthologous encoding key enzymes in TAG biosynthetic pathway

	Enzyme name	Gene copy number		
		Arabidopsis^1^	Cocoa^1^	Shea^2^
ACC2	Homomeric Acetyl-CoA Carboxylase	1	1	1
CAC2	Homomeric Acetyl-CoA Carboxylase BC subunit	1	1	1
BCCP(CAC1A)	Homomeric Acetyl-CoA Carboxylase BCCP subunit	2	3	1
CAC3	Homomeric Acetyl-CoA Carboxylase alpha-CT subunit	1	2	2
ACCD	Homomeric Acetyl-CoA Carboxylase beta-CT subunit	1	1	1
MAT	Plastidial malonyl-CoA: ACP Malonyltransferase	1	1	1
KAS I	Ketoacyl-ACP synthase I	1	2	4
KAS II	Ketoacyl-ACP synthase II	1	3	4
KAS III	Ketoacyl-ACP synthase III	1	1	2
KAR	Plastidial ketoacyl-ACP Reductase	5	3	5
HAD	Plastidial Hydroxyacyl-ACP Dehydrase	2	1	2
ENR1	Plastidial Enoyl-ACP Reductase	1	2	2
FAB2	Stearoyl-ACP Desaturase	7	8	2
ACP	Plastidial Acyl Carrier Protein	5	3	3
ACP	Mitochondrial Acyl Carrier Protein	3	4	5
FATA	Acyl-ACP Thioesterase Fat A	2	1	2
FATB	Acyl-ACP Thioesterase Fat B	1	5	3
FAD2	ER Oleate Desaturase	1	2	2
FAD3	ER Linoleate Desaturase	1	1	2
FAD4	Phosphatidylglycerol Desaturase	1	1	1
FAD5	Monogalactosyldiacylglycerol Desaturase	1	3	2
FAD6	Plastidial Oleate Desaturase	1	1	1
FAD7/8	Platidial Linoleate Desaturase	2	2	2
KCS	beta-Ketoacyl-CoA synthase	21	20	5
KCR	Ketoacyl-CoA Reductase	2	2	2
ECR	Enoyl-CoA Reductase	1	1	1
LACS	Long Chain Acyl-CoA Synthetase	2	7	5
GPAT	glycerol-3-phosphate acyltransferase	10	13	4
LPAT	lysophosphatidic acid acyltransferase	9	10	8
PAP	Phosphatidic acid phosphatase	2	2	2
DGAT	Acyl-CoA:Diacylglycerol acyltransferase	3	2	3

The incorporation of acyl moieties into TAGs is catalyzed by three different kinds of enzymes, GPAT, LPAT, and DGAT, which can add acyl-coenzyme As (acyl-CoA) to the *sn*-1, *sn*-2 and *sn*-3 position of glycerol, respectively (Chapman and Ohlrogge 2012). In total, 4 full-length GPAT genes named *VpGPAT1* to *VpGPAT4*, 8 full-length LPAT genes named *VpLPAT1* to *VpLPAT8*, and 11 full-length DGAT genes named *VpDGAT1* to *VpDGAT11* were predicted from the shea tree transcriptome data (Table 1). Some shea tree GPAT, LPAT, and DGAT genes showed high identities with known cocoa GPAT, LPAT, and DGAT genes (Figure S3a–c), such as the identity between *VpGPAT2* and *TcGPAT9* was 85% and the identity between *VpDGAT7* and *TcDGAT2* was 69.7%. The expression levels of most GPAT, LPAT, and DGAT genes were similar in sample T3 and T6 (Table S5).

### Characterization of cloned TAG biosynthetic genes

**Fig. 3:**
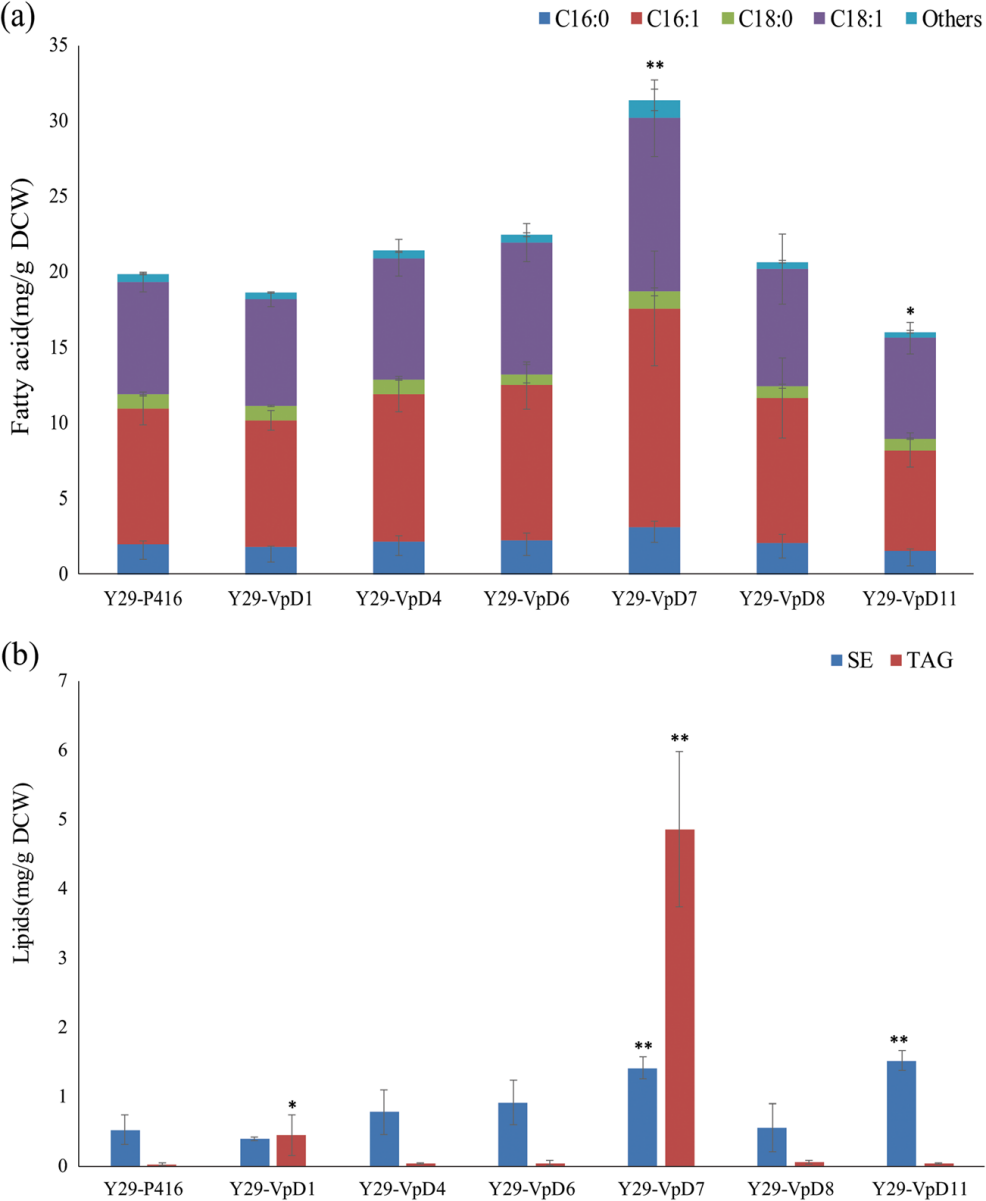
Total fatty acid (**a**) and lipid (**b**) production of *S. cerevisiae* Y29 strains harboring empty plasmid or plasmid harboring shea genes. SE, steryl esters. Asterisks (*) indicate significant differences of fatty acids (a) and lipid (b) between *S. cerevisiae* Y29 strains harboring empty plasmid and *S. cerevisiae* Y29 strains harboring shea genes; “*” indicates *p* < 0.05; “**” indicates *p* < 0.01. The *p* values are calculated based on paired *t* tests corrected for multiple comparisons

A total of 3 potential GPAT, 5 potential LPAT, and 6 potential DGAT genes of shea tree were cloned from shea tree cDNA and their functions were investigated. As described in our previous study, it is hard to replace yeast GPAT or LPAT genes with plant GPAT or LPAT genes, whereas it is possible to verify the potential functions of DGAT genes by using DGAT-deficient yeast strains (Wei et al. 2018). We expressed 7 potential shea DGAT genes in the DGAT-deficient yeast strain Y29 (IMX581 sct1Δ ale1Δ lro1Δ dga1Δ). Y29-VpD7 harboring *VpDGAT7* showed a significant increase in total fatty acid production over the control strain, indicating *VpDGAT7* was functionally expressed in yeast and might be functioned as DGAT in shea tree (Fig. 3a) (Wei et al. 2018). The expression of *VpDGAT1* or *VpDGAT7* in Y29 strain led to a significant increase over control and recovered TAG production of Y29 (Fig. 3b).

**Fig. 4:**
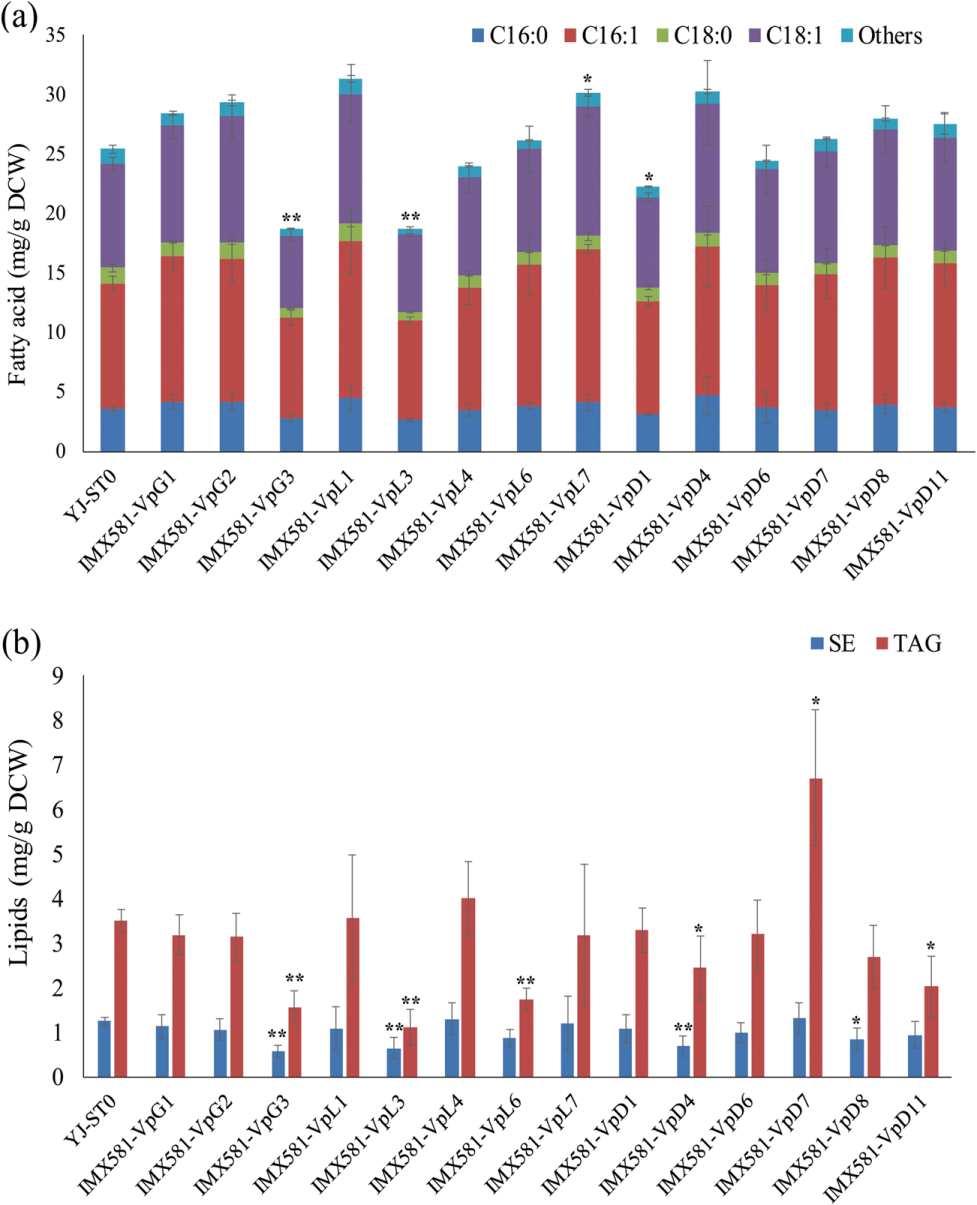
Total fatty acid (**a)** and neutral lipid (**b**) production in YJ-ST0 and *S. cerevisiae* strains harboring shea genes. Others represent the summed content of C12:0, C14:0, C14:1, C20:0, C20:1, C22:0, C24:0, and C26:0 fatty acids. The error bars represent the standard deviation of three biological replicates. Asterisks (*) indicate significant difference between the yeast strains harboring shea genes and YJ-ST0. “*” indicates *p* < 0.05; “**” indicates *p* < 0.01. The *p* values are calculated based on paired *t* tests corrected for multiple comparisons

Moreover, all the cloned shea GPAT, LPAT, and DGAT genes were expressed in wild-type yeast strains. Fatty acid production and lipid production of the heterogeneous expression strains harboring the cloned shea genes were changed (Fig. 4). The strain with *VpLPAT7* overexpression showed a significant increase in total fatty acid production compared with control (Fig. 4a). The strain harboring *VpDGAT7* showed a significant increase in total TAG production over control (Fig. 4b). However, the expression of other cloned shea GPAT, LPAT, and DGAT genes individually in yeast is consistent with our earlier study on expressing cocoa genes in yeast that single expression of mostcocoa TAG biosynthetic genes did not increase total lipid production over the control strain (Fig. 4b) (Wei et al. 2018).

### TAG profiles of different yeast strains harboring shea genes

**Fig. 5:**
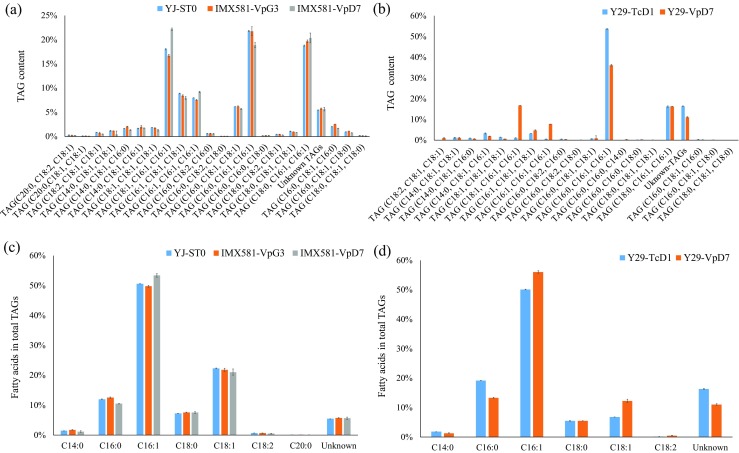
Relative TAG content and relative TAG fatty acid composition of *S. cerevisiae* strains. **a** Relative TAG content (Area%) of different *S. cerevisiae* IMX581-derived strains. **b** Relative TAG content (Area%) of different *S. cerevisiae* Y29-derived strains. **c** Relative fatty acid composition (Area%) of the TAGs of *S. cerevisiae* IMX581-derived strains. **d** Relative fatty acid composition (Area%) of the TAGs of *S. cerevisiae* Y29–derived strains. The error bars represent the standard deviation of two biological replicates. Asterisks (*) indicate a significant difference between the yeast strains harboring shea genes and YJ-ST0. “*” indicates *p* < 0.05; “**” indicates *p* < 0.01. The *p* values are calculated based on paired *t* tests corrected for multiple comparisons

IMX581-VpG3 showed significantly decreased fatty acid and lipid production, while IMX581-VpD7 showed significantly increased lipid production compared with the control strain (Fig. 4b). TAG profiles of IMX581-VpG3 and the control strain YJ-ST0 were similar, but IMX581-VpD7 had a different profile compared with IMX581-VpG3 and YJ-ST0 (Fig. 5a). For instance, compared with IMX581-VpG3 and YJ-ST0, TAG (C18:1, C16:1, C16:1) of IMX581-VpD7 increased, while TAG (C16:0, C16:1, C16:1) of IMX581-VpD7 decreased. Consequently, C16:0 decreased and C16:1 increased in the TAGs of IMX581-VpD7 (Fig. 5c). However, the expression of these two shea tree genes did not have significant influence in TAG fatty acid distribution in yeast (Fig. 5c).

We also compared the TAG profiles of Y29-VpD7 against Y29-TcD1 which harbored cocoa *TcDGAT1* gene, showing they had different TAG profiles and contents (Fig. 5b) (Wei et al. 2018). Y29-VpD7 accumulated some TAGs at a higher proportion than Y29-TcD1, such as TAG (C18:1, C16:1, C16:1) and TAG (C16:1, C16:1, C16:1), and contained less TAG (C16:0, C16:1, C16:1) than Y29-TcD1 (Fig. 5b). However, SOS composition in Y29-VpD7 was not high (Fig. 5b). Though C18:1 in TAGs of Y29-VpD7 was more abundant than Y29-TcD1, unknown fatty acids of Y29-VpD7 was less abundant than Y29-TcD1 (Fig. 5d).

## Discussion

Though shea tree is a major agroforestry tree in Africa, and its butter can be used as raw materials in food and cosmetics industries (Jahurul et al. 2013), little is known about its functional genes (Abdulai et al. 2017; Allal et al. 2008; Di Vincenzo et al. 2005; Fontaine et al. 2004; Kelly et al. 2004). Recently, omics analyses had made great advances in plant functional gene recovery (Gomes de Oliveira Dal’Molin and Nielsen 2018; Yao et al. 2016). In this study, we analyzed seven shea fruits picked in Africa. Further sequencing transcriptomics of two shea fruits recovered some potential TAG biosynthetic genes. The overexpression of some genes cloned from shea fruit cDNA in yeast changed yeast lipid profiles and identified some functional TAG biosynthetic genes of shea tree.

Previous studies have suggested that the abundance of C18:0 and C18:1 in cocoa TAGs increases, while the abundance of C16:0 and C18:2 decreases during cocoa bean ripe process (Patel and Shanklin 1994; Zhang et al. 2015). The lipid variation of shea fruit samples T1 to T7 was similar with the corresponding lipid profiles reported in cocoa bean ripe processes, showing T1 to T7 can represent different stages during shea fruits ripeness (Fig. 1a–c and Figure S2) (Patel and Shanklin 1994). Besides, different SOO and SOS compositions in shea fruits suggested T2–T3 and T4–T7 might represent different SOO- and SOS-containing shea fruits (Fig. 1c), and sequencing T3 and T6 would reveal most functional genes in shea tree. Though it should be mentioned here that the identified shea genes were only based on transcriptome data and there may even more genes associated with lipid metabolism in the shea tree genome, suggesting there might be more TAG biosynthetic genes in shea tree than *A*. *thaliana* and cocoa tree.

GPAT, LPAT, and DGAT are important for CB or CBS (SOS) production in plants (Xu and Shanklin 2016). A previous study had revealed that several cocoa genes functioned and was helpful for yeast CBS production (Wei et al. 2018; Wei et al. 2017a). Mainly, a single expression of some cocoa GPAT, LPAT, or DGAT genes could significantly increase lipid production in yeast, and a single overexpression of some shea genes had similar results. However, SOS and C18 fatty acid production in wild-type yeast harboring shea genes did not increase, hinting that the GPAT, LPAT, and DGAT genes of shea tree should be constitutively expressed to produce shea butter (SOS). Overexpression of *VpGPAT3* or *VpDGAT7* affected yeast lipid production, suggesting that they functioned in yeast and might participate in shea butter biosynthesis. Expression genes of *VpDGAT1* or *VpDGAT7* in Y29 recover TAG production ability of Y29, indicating *VpDGAT1* and *VpDGAT7* were the functional DGAT genes in TAG production of shea tree. Besides, different distributions of TAG fatty acids in Y29-TcD1 and Y29-VpD7 suggested that the shea DGATs might have a different substrate specificity compared with the cocoa DGAT previously expressed in *S. cerevisiae* for TAG production (Wei et al. 2018; Wei et al. 2017a). Unfortunately, SOS production was not high in Y29-VpD7 and IMX581-VpD7, and future co-expression of GPAT, LPAT, and DGAT genes of shea tree in yeast might increase yeast SOS production (Wei et al. 2018; Wei et al. 2017a).

In summary, we measured lipid content, composition, and TAG composition of seven shea fruits harvested in Africa and performed transcriptome analysis on two fruits. The transcriptome analysis revealed a list of genes potentially involved in TAG biosynthesis and their respective transcription levels in the two obviously different fruits. It also revealed that the shea tree genome encodes a high number of lipid biosynthetic genes, such as ketoacyl-ACP synthase genes. This might be one of the reasons for the high lipid content of shea fruits. Further heterologous expression of GPAT, LPAT, and DGAT genes in yeast identified several functional shea tree TAG biosynthetic genes. As yeast can be modulated for SOS lipid production and reprogramming of cellular metabolism pathway could turn *S. cerevisiae* to an oleaginous yeast (Bergenholm et al. 2018; Yu et al. 2018), further expression of the identified TAG biosynthetic genes in the engineered yeast might be used for microbial SOS (shea butter) production.

## Electronic supplementary material

ESM 1 (PDF 367 kb)
