# Supplementary material for: Identification of genes involved in shea butter biosynthesis from Vitellaria paradoxa fruits through transcriptomics and functional heterologous expression
Source: Appl Microbiol Biotechnol. 2019 Mar 26;103(9):3727–36. doi: 10.1007/s00253-019-09720-3 (PMC6469615; doi:10.1007/s00253-019-09720-3)
Supplement: Supplementary file 1 — (PDF 367 kb) [file 253_2019_9720_MOESM1_ESM.pdf]

Applied Microbiology and Biotechnology

Supplementary information

Identification of genes involved in shea butter biosynthesis from *Vitellaria paradoxa* fruits through transcriptomics and functional heterologous expression

Yongjun Wei[1,2,3], Boyang Ji[2,3], Verena Siewers[2,3] Deyang Xu[4], Barbara Ann Halkier[4], and Jens Nielsen[2,3,5*]

[1]School of Pharmaceutical Sciences, Key Laboratory of State Ministry of Education, Key Laboratory of Henan province for Drug Quality Control and Evaluation, Collaborative Innovation Center of New Drug Research and Safety Evaluation, Zhengzhou University, 100 Kexue Avenue, Zhengzhou, Henan, 450001, China.
[2]Department of Biology and Biological Engineering, Chalmers University of Technology, SE-41296 Gothenburg, Sweden
[3]Novo Nordisk Foundation Center for Biosustainability, Chalmers University of Technology, SE-41296 Gothenburg, Sweden
[4]DynaMo Center, Department of Plant and Environmental Sciences, University of Copenhagen, Thorvaldsensvej 40, 1871 Frederiksberg C, Denmark
[5]Novo Nordisk Foundation Center for Biosustainability, Technical University of Denmark, DK-2800 Kgs. Lyngby, Denmark

[*]Correspondening author:
Email: Jens Nielsen (nielsenj@chalmers.se)
TEL: +46 (0)31 772 3804

The supplementary information contains 3 figures and 5 tables.

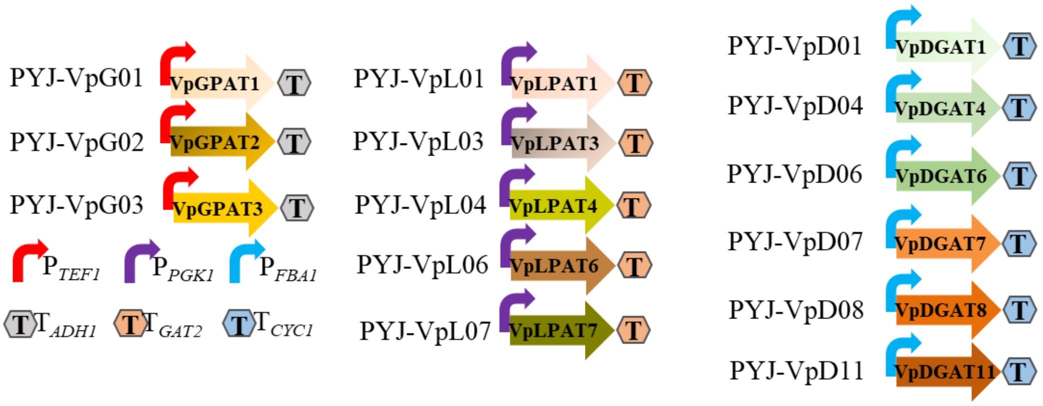

Figure S1 Schematic organization of shea tree gene expression cassettes in each of the expression plasmids.

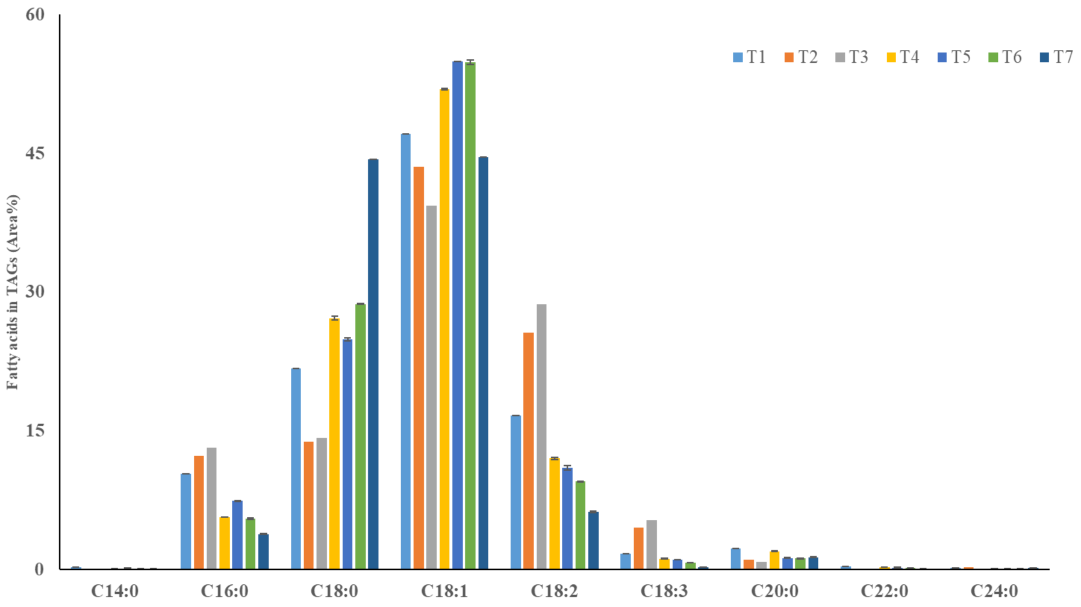

Figure S2 Relative fatty acid composition of the TAGs in the 7 shea fruits. The error bars of fruit T4 to T7 represented two technically replicates.

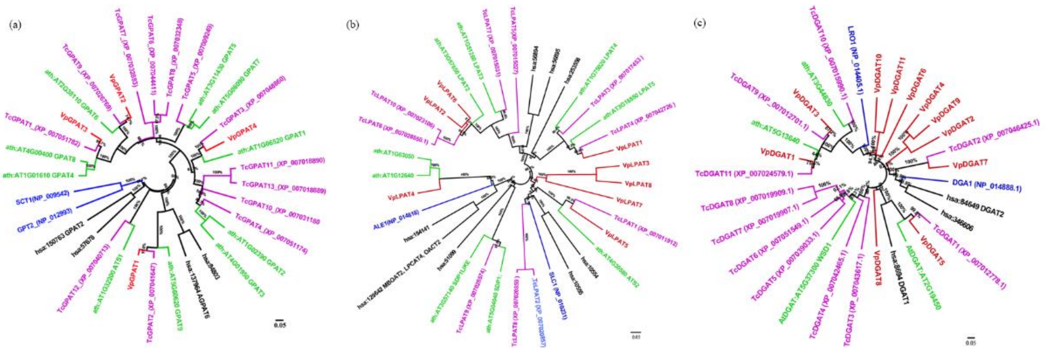

Figure S3 Phylogenetic analysis of GPAT (a), LPAT (b) and DGAT (c) genes with an unrooted tree. The tree was constructed using the MEGA 7.0.21 software (bootstrap values: 1000) with the peptide sequences, and the method was neighbor-joining method. Shea genes are marked with red; yeast genes are marked with blue; Cocoa genes are marked with purple; A. thaliana genes are marked with green; H. sapiens genes are marked with black. The bootstrap values are marked above the nodes and the scale bar is indicated under each tree.

Table S1 Weight of the 7 shea fruits.

Fruit name	Collection date	Shea fruit weight (g)
T1	15-Apr-15	9.56
T2	24-Apr-15	8.1
T3	29-Apr-15	8.42
T4	6-May-15	7.56
T5	13-May-15	11.32
T6	21-May-15	12.06
T7	15-Jun-15	8.08

Table S2 Strains used in this study

Strains	Parent strains	Expression plasmids	Properties
YJ-ST0	IMX581	PRS416	Empty plasmid pRS416
IMX581-VpG1	IMX581	PYJ-VpG1	VpGPAT1 expression from pRS416 plasmid
IMX581-VpG2	IMX581	PYJ-VpG2	VpGPAT2 expression
IMX581-VpG3	IMX581	PYJ-VpG3	VpGPAT3 expression
IMX581-VpL1	IMX581	PYJ-VpL1	VpLPAT1 expression
IMX581-VpL3	IMX581	PYJ-VpL3	VpLPAT3 expression
IMX581-VpL4	IMX581	PYJ-VpL4	VpLPAT4 expression
IMX581-VpL6	IMX581	PYJ-VpL6	VpLPAT6 expression
IMX581-VpL7	IMX581	PYJ-VpL7	VpLPAT7 expression
IMX581-VpD1	IMX581	PYJ-VpD1	VpDGAT1 expression
IMX581-VpD4	IMX581	PYJ-VpD4	VpDGAT4 expression
IMX581-VpD6	IMX581	PYJ-VpD6	VpDGAT6 expression
IMX581-VpD7	IMX581	PYJ-VpD7	VpDGAT7 expression
IMX581-VpD8	IMX581	PYJ-VpD8	VpDGAT8 expression
IMX581-VpD11	IMX581	PYJ-VpD11	VpDGAT11 expression
Y29	IMX581	None	sct1Δ ale1Δ lro1Δ dga1Δ
Y29-P416	Y29	PRS416	Empty plasmid PRS416
Y29-VpD1	Y29	PYJ-VpD1	VpDGAT1 expression
Y29-VpD4	Y29	PYJ-VpD4	VpDGAT4 expression
Y29-VpD6	Y29	PYJ-VpD6	VpDGAT6 expression
Y29-VpD7	Y29	PYJ-VpD7	VpDGAT7 expression
Y29-VpD8	Y29	PYJ-VpD8	VpDGAT8 expression
Y29-VpD11	Y29	PYJ-VpD11	VpDGAT11 expression

Table S3 Primers used for gene cloning and expression.

**Name**	**Sequences (5'-3')**	**Application**
VpGPAT1-fw	CGTTAGAAGAAGTAAGAACC	Shea gene cloning
VpGPAT1-rv	TAAGGAAAATATTGTCAACA	Shea gene cloning
VpGPAT2-fw	CCCTTCTCTCTCTATATTTC	Shea gene cloning
VpGPAT2-rv	TAATTTCTTCCGTTAATTAA	Shea gene cloning
VpGPAT3-fw	GTTTTTCTTCTCTCTCTTTG	Shea gene cloning
VpGPAT3-rv	TTATCAATCGTCTTTCTGATGCAAAAC	Shea gene cloning
VpGPAT4-fw	ATATTGATAGCGTGCCAGA	Shea gene cloning
VpGPAT4-rv	GTGAATTAGGGTTTAACTTT	Shea gene cloning
VpLPAT1-fw	TCTGGAAGGTAGACTGTATC	Shea gene cloning
VpLPAT1-rv	TAGGTAACGCATAAAAATAC	Shea gene cloning
VpLPAT2-fw	CTCCCTTGTTTTCTCTG	Shea gene cloning
VpLPAT2-rv	GCTACAAGAACTTGAGAACT	Shea gene cloning
VpLPAT3-fw	ACCTCTCCACCTCCTCCTTC	Shea gene cloning
VpLPAT3-rv	AAAAGAAGAGAGAGGCCCGC	Shea gene cloning
VpLPAT4-fw	ATCCAATCTCCACTTTCT	Shea gene cloning
VpLPAT4-rv	TCTAAATTTTGACAAATTCA	Shea gene cloning
VpLPAT5-fw	GCTTAGAGAGACAGAGAGAG	Shea gene cloning
VpLPAT5-rv	CAAACTTAATACAGCATACC	Shea gene cloning
VpLPAT6-fw	CCCTGTCTTTCGCGTTCGTA	Shea gene cloning
VpLPAT6-rv	TTTGACTGGTGGGCAGTCAA	Shea gene cloning
VpLPAT7-fw	GTTTTTCTTCTCTCTCTTTG	Shea gene cloning
VpLPAT7-rv	GACACATCATAGCACATTAA	Shea gene cloning
VpLPAT8-fw	TTCTTCTCTTCGCGGCCTTC	Shea gene cloning
VpLPAT8-rv	CCACAAACCTCTCCCTCAGC	Shea gene cloning
VpDGAT1-fw	CAAGTTACATGCATAAAAAT	Shea gene cloning
VpDGAT1-rv	CAATTACTTCGAATGAATAA	Shea gene cloning
VpDGAT2-fw	TGAGTTGCTTACAGTTACAG	Shea gene cloning
VpDGAT2-rv	TACAAGCAAATTACAGTCAC	Shea gene cloning
VpDGAT3-fw	TTTAAAGAGCTTCATTTACA	Shea gene cloning
VpDGAT3-rv	TAGAGTAACAAAAATTCACG	Shea gene cloning
VpDGAT4-fw	CGAGAAGAAGCAAGAGG	Shea gene cloning
VpDGAT4-rv	ACTCATACTCCAGAGGTAAA	Shea gene cloning
VpDGAT5-fw	TTATCTCCTCTTCTTTTTTTGC	Shea gene cloning
VpDGAT5-rv	TTTTCCACTAACAAATTAAA	Shea gene cloning
VpDGAT6-fw	TTTACTTTCACAAACTGAAG	Shea gene cloning
VpDGAT6-rv	TTACAGCAATTTTACAGTGT	Shea gene cloning
VpDGAT7-fw	AGACCATAAACTATTTGGAT	Shea gene cloning
VpDGAT7-rv	TTAATTTACACTCAAATCCA	Shea gene cloning
VpDGAT8-fw	AAAATCCCATTTATCAATAT	Shea gene cloning
VpDGAT8-rv	AACATCAGAAGACGTACTGT	Shea gene cloning
VpDGAT9_fw	ATGGCCACGAACGACGGCGGAC	Shea gene cloning
VpDGAT9_rv	TCAAAATTCAAATGTTGGGACTTCGC	Shea gene cloning
VpDGAT10_fw	ATGACGGTGTTGTTGGGGGACATAG	Shea gene cloning
VpDGAT10_rv	TCAGGCAGTGGCAGCCACACTC	Shea gene cloning
VpDGAT11_fw	ATGGTCGTCGGAGACTGGTGTTGC	Shea gene cloning
VpDGAT11_rv	TTAATCTATAAATTTAACATCCATCACTTTGG	Shea gene cloning
PRS-416 backbone_F1	CTTCCGGCTGGCTGGTTTATTG	Plasmid amplification
PRS-416 backbone_R1	GAGCTCCAGCTTTTGTTCCCTTTAGTG	Plasmid amplification
PRS-416 backbone_F2	CTGAAAACCTTGCTTGAGAAGGTTTTG	Plasmid amplification

PRS-416 backbone_R2	CCGGCTCCAGATTTATCAGC	Plasmid amplification
pTEF1_fw	ACGCCAAGCGCGCAATTAACCCTCACTAAAGGGAACAAAAGCTGGAGCTCATAGCTTCAAAATGTTTCTACTCCTTTTTTAC	Promoter amplification
pTEF1_rv	TTTGTAATTAAAACTTAGATTAGATTGC	Promoter amplification
tADH1_fw	GCGAATTTCTTATGATTTATGATTTTTATT	Terminator amplification
tADH1_rv	CAAATTAAAGCCTTCGAGCGTCCCAAAACCTTCTCAAGCAAGGTTTTCAGTTGCTCGGCATGCCGGTAGAGG	Terminator amplification
pPGK1_fw	ACGCCAAGCGCGCAATTAACCCTCACTAAAGGGAACAAAAGCTGGAGCTCGAAGTACCTTCAAAGAATGGGGTC	Promoter amplification
pPGK1_rv	TTTGTTATATTTGTTGTAAAAAGTAGATAAT	Promoter amplification
tGAT2_fw	TATAAAATCATACATTCATATAATATCCAT	Terminator amplification
tGAT2_rv	CAAATTAAAGCCTTCGAGCGTCCCAAAACCTTCTCAAGCAAGGTTTTCAGGGGAAAACGTTAGGAAAACGCGG	Terminator amplification
pFBA1_fw	ACGCCAAGCGCGCAATTAACCCTCACTAAAGGGAACAAAAGCTGGAGCTCTCCAACTGGCACCGCTGGC	Promoter amplification
pFBA1_rv	TTTGAATATGTATTACTTGGTTATGG	Promoter amplification
tCYC_rv	ACAGGCCCCTTTTCCTTTGTC	Terminator amplification
tCYC1_rv	CAAATTAAAGCCTTCGAGCGTCCCAAAACCTTCTCAAGCAAGGTTTTCAGTATAATGTTACATGCGTACACGCG	Terminator amplification
VpGPAT1_pTEF1_fw	ATCTAATCTAAGTTTTAATTACAAAATGAGTGAATTGAACCCCTCG	Shea gene expression
VpGPAT1_tADH1_rv	AAATCATAAATCATAAGAAATTCGCTTATTTTTCCTCCAGGCGACGTAG	Shea gene expression
VpGPAT2_pTEF1_fw	ATCTAATCTAAGTTTTAATTACAAAATGGGAAGGTATCGCCCTTTTG	Shea gene expression
VpGPAT2_tADH1_rv	AAATCATAAATCATAAGAAATTCGCTCAATCCTTCTCCTTCACCTCC	Shea gene expression
VpGPAT3_pTEF1_fw	ATCTAATCTAAGTTTTAATTACAAAATGTCGCCGGCGAAGCCATCC	Shea gene expression
VpGPAT3_tADH1_rv	AAATCATAAATCATAAGAAATTCGCTCACTTCTTGGAATACATAGATTCCAC	Shea gene expression
VpLPAT1_pPGK1_fw	TACTTTTTACAACAAATATAACAAAATGGAGGGAGATGCAAGTTCG	Shea gene expression
VpLPAT1_tGAT2_rv	TATTATATGAATGTATGATTTTATATCACGCACCCAACAATGGGC	Shea gene expression
VpLPAT4_pPGK1_fw	TACTTTTTACAACAAATATAACAAAATGCTTCTTGGCTACGCCTCAATG	Shea gene expression
VpLPAT4_tGAT2_rv	TATTATATGAATGTATGATTTTATATCACTGTTCTTTCTGAGCTTTAGATC	Shea gene expression
VpLPAT6_pPGK1_fw	TACTTTTTACAACAAATATAACAAAATGGCGGTTCCAGCGGCAGTCG	Shea gene expression
VpLPAT6_tGAT2_rv	TATTATATGAATGTATGATTTTATACTACTGTTTTTTGTCTGGTCCTGTCTC	Shea gene expression

VpLPAT7_pPGK1_fw	TACTTTTTACAACAAATATAACAAAATGGAGTCCGAACTCAAGGAG	Shea gene expression
VpLPAT7_tGAT2_rv	TATTATATGAATGTATGATTTTATATCAATCGTCTTTCTGATGCAAAAC	Shea gene expression
VpDGAT1_pFBA1_fw	CATAACCAAGTAATACATATTCAAAATGCCTTTGATTCGACGCAAAAAG	Shea gene expression
VpDGAT1_tCYC1_rv	TATCGACAAAGGAAAAGGGGCCTGTCTACAGTTGTAAGTTGATCTTGTC	Shea gene expression
VpDGAT4_pFBA1_fw	CATAACCAAGTAATACATATTCAAAATGGCGAAACACGAAGGTGAG	Shea gene expression
VpDGAT4_pFBA1_rv	TATCGACAAAGGAAAAGGGGCCTGTTTAAATATCAAACGTTGGTACTTCAG	Shea gene expression
VpDGAT6_pFBA1_fw	CATAACCAAGTAATACATATTCAAAATGGGTAATAATGTCTTCCGTTAC	Shea gene expression
VpDGAT6_pFBA1_rv	TATCGACAAAGGAAAAGGGGCCTGTTTATACATAATGTAAGAGGAGATCAGC	Shea gene expression
VpDGAT7_pFBA1_fw	CATAACCAAGTAATACATATTCAAAATGGCCACGGAGCAGGCTAAC	Shea gene expression
VpDGAT7_tCYC1_rv	TATCGACAAAGGAAAAGGGGCCTGTTCAGAGAATTCTCAGTTGAAGATTC	Shea gene expression
VpDGAT8_pFBA1_fw	CATAACCAAGTAATACATATTCAAAATGAACAACAGTAGCTCTTGGC	Shea gene expression
VpDGAT8_tCYC1_rv	TATCGACAAAGGAAAAGGGGCCTGTCTAATGTCTAGTCCCTCTTCTTTGC	Shea gene expression
VpDGAT11_pFBA1_fw	CATAACCAAGTAATACATATTCAAAATGGTCGTCGGAGACTGGTGTTGC	Shea gene expression
VpDGAT11_tCYC1_rv	TATCGACAAAGGAAAAGGGGCCTGTTTAATCTATAAATTTAACATCCATCACTTTGG	Shea gene expression
VpGPAT2 sequence primer	CCAGATTCTACGCCGAGGAC	Sequencing primer
VpDGAT4 sequence primer	TTACTTTGCTCCAGGCTATTTCG	Sequencing primer
VpDGAT8 sequence primer	ATCTACACGGAGCTTGTGCC	Sequencing primer
DGA1_targetRNA fw	TGCGCATGTTTCGGCGTTCGAAACTTCTCCGCAGTGAAAGATAAATGATCAAATGATTAACAACATCATCGTTTTAGAGCTAGAAATAGCAAGTTAAAATAAGGCTAGTCCGTTATCAAC	gRNA cassette construction
DGA1_targetRNA rv	GTTGATAACGGACTAGCCTTATTTTAACTTGCTATTTCTAGCTCTAAAACGATGATGTTGTTAATCATTTGATCATTTATCTTTCACTGCGGAGAAGTTTCGAACGCCGAAACATGCGCA	gRNA cassette construction
DGA1_repair oligo fw	CACATACACTTACATATACATAAGGAAACGCAGAGGCATACAGTTTGAACAGTCACATAATAATGAATTCATTGGAAAACACAAAATATGTTAGAATAAATAAGGATTTTTTAGTGTTTG	Repair oligos
DGA1_repair oligo rv	CAAACACTAAAAAATCCTTATTTATTCTAACATATTTTGTGTTTTCCAATGAATTCATTATTATGTGACTGTTCAAACTGTATGCCTCTGCGTTTCCTTATGTATATGTAAGTGTATGTG	Repair oligos
DGA1_dg fw	GAAGTACTTCACCACGGGGG	Diagnostic primers for seamless deletion confirmation

DGA1_dg rv	GCCTCTCAGTTACGCTTTGC	Diagnostic primers for seamless deletion confirmation
LRO1_targetRNA fw	TGCGCATGTTTCGGCGTTCGAAACTTCTCCGCAGTGAAAGATAAATGATCGATTCTGATGAAAACAATAAGTTTTAGAGCTAGAAATAGCAAGTTAAAATAAGGCTAGTCCGTTATCAAC	gRNA cassette construction
LRO1_targetRNA rv	GTTGATAACGGACTAGCCTTATTTTAACTTGCTATTTCTAGCTCTAAAACTTATTGTTTTCATCAGAATCGATCATTTATCTTTCACTGCGGAGAAGTTTCGAACGCCGAAACATGCGCA	gRNA cassette construction
LRO1_repair oligo fw	ATAGTAACAGCCATTACAAAAGGTTCTCTACCAACGAATTCGGCGACAATCGAGTAAAAAATGACCGACATTGACTCACTATCCATCCGTGTATTATTTCAAAGAGCGAAAAGAAGGCGC	Repair oligos
LRO1_repair oligo rv	GCGCCTTCTTTTCGCTCTTTGAAATAATACACGGATGGATAGTGAGTCAATGTCGGTCATTTTTTACTCGATTGTCGCCGAATTCGTTGGTAGAGAACCTTTTGTAATGGCTGTTACTAT	Repair oligos
LRO1_dg fw	TCTGGCCCTTCAACTTCGAC	Diagnostic primers for seamless deletion confirmation
LRO1_dg rv	GTACGTCGCTGCTGCAATTG	Diagnostic primers for seamless deletion confirmation
SCT1_targetRNA fw	TGCGCATGTTTCGGCGTTCGAAACTTCTCCGCAGTGAAAGATAAATGATCCAAGTCTACAGTTAAAATAAGTTTTAGAGCTAGAAATAGCAAGTTAAAATAAGGCTAGTCCGTTATCAAC	gRNA cassette construction
SCT1_targetRNA rv	GTTGATAACGGACTAGCCTTATTTTAACTTGCTATTTCTAGCTCTAAAACTTATTTTAACTGTAGACTTGGATCATTTATCTTTCACTGCGGAGAAGTTTCGAACGCCGAAACATGCGCA	gRNA cassette construction
SCT1_repair oligo fw	GCCCGGAATTAAATATATAGTAAAAAGAGCACAGGGGCGTTTACATCGGGGTAAAAAAAAATGCCATTTACTGACGGTGAAGATACTAGAAACTAAATCTTTCGCCGTTCTATTTATGTA	Repair oligos
SCT1_repair oligo rv	TACATAAATAGAACGGCGAAAGATTTAGTTTCTAGTATCTTCACCGTCAGTAAATGGCATTTTTTTTTACCCCGATGTAAACGCCCCTGTGCTCTTTTTACTATATATTTAATTCCGGGC	Repair oligos
SCT1_dg fw	AAGTAGGCCCGCCTTCTTTC	Diagnostic primers for seamless deletion confirmation
SCT1_dg rv	CGGCTACTGGTCTCTTCCTG	Diagnostic primers for seamless deletion confirmation
SLC4_targetRNA fw	TGCGCATGTTTCGGCGTTCGAAACTTCTCCGCAGTGAAAGATAAATGATCTATTTACGTGTCACAAAGAAGTTTTAGAGCTAGAAATAGCAAGTTAAAATAAGGCTAGTCCGTTATCAAC	gRNA cassette construction

SLC4_targetRNA rv	GTTGATAACGGACTAGCCTTATTTTAACTTGCTATTTCTAGCTCTAAAACTTCTTTGTGACACGTAAATAGATCATTTATCTTTCACTGCGGAGAAGTTTCGAACGCCGAAACATGCGCA	gRNA cassette construction
SLC4_repair oligo fw	CAAACCGCATACGCCAAGACAAACCGTGGTGATTTAATTCTGCTGCTGATCGCTTCCAACATGGCCAAAAACGACAGATGCGTGTGGAAGTCACAGTCTTGTTGTCTTATTTTCCAGTTC	Repair oligos
SLC4_repair oligo rv	GAACTGGAAAATAAGACAACAAGACTGTGACTTCCACACGCATCTGTCGTTTTTGGCCATGTTGGAAGCGATCAGCAGCAGAATTAAATCACCACGGTTTGTCTTGGCGTATGCGGTTTG	Repair oligos
SLC4_dg fw	GTCGAAACCACAAACCGTCG	Diagnostic primers for seamless deletion confirmation
SLC4_dg rv	ACCGTACCGCCATCTCAAAG	Diagnostic primers for seamless deletion confirmation
GPT2_targetRNA fw	TGCGCATGTTTCGGCGTTCGAAACTTCTCCGCAGTGAAAGATAAATGATCAATTATTCACTTAAAAAAACGTTTTAGAGCTAGAAATAGCAAGTTAAAATAAGGCTAGTCCGTTATCAAC	gRNA cassette construction
GPT2_targetRNA rv	GTTGATAACGGACTAGCCTTATTTTAACTTGCTATTTCTAGCTCTAAAACGTTTTTTTAAGTGAATAATTGATCATTTATCTTTCACTGCGGAGAAGTTTCGAACGCCGAAACATGCGCA	gRNA cassette construction
GPT2_repair oligo fw	AATTCTACTTTCACATCTAATAAAGCCGATTAATCGATCAGTTATTGCTCCCTTTCCTTTTCAAAAAATAGAAAAATAAAAAAAAGCATTTGACAATGTTTGTAAATAATATTATGAAAG	Repair oligos
GPT2_repair oligo rv	CTTTCATAATATTATTTACAAACATTGTCAAATGCTTTTTTTTATTTTTCTATTTTTTGAAAAGGAAAGGGAGCAATAACTGATCGATTAATCGGCTTTATTAGATGTGAAAGTAGAATT	Repair oligos
GPT2_dg fw	TTCGTGCTTCGTCGCTAGAG	Diagnostic primers for seamless deletion confirmation
GPT2_dg rv	TCTTGGCTAGGACGGCATTG	Diagnostic primers for seamless deletion confirmation
SLC1_targetRNA fw	TGCGCATGTTTCGGCGTTCGAAACTTCTCCGCAGTGAAAGATAAATGATCTAACATGAAGATATCCAAGGGTTTTAGAGCTAGAAATAGCAAGTTAAAATAAGGCTAGTCCGTTATCAAC	gRNA cassette construction
SLC1_targetRNA rv	GTTGATAACGGACTAGCCTTATTTTAACTTGCTATTTCTAGCTCTAAAACCCTTGGATATCTTCATGTTAGATCATTTATCTTTCACTGCGGAGAAGTTTCGAACGCCGAAACATGCGCA	gRNA cassette construction
SLC1_repair oligo fw	AATTCTTCAATAGAGAAGTTTAGTGGTTTCCCTCCGTCAGTGAATTCGAGCAAAAAAATAGCCACCACCACATTTTTAGAGTAGTATATAGACCCAAAAACTGTAATTATCTTTTTAAAA	Repair oligos

SLC1_repair oligo rv	TTTTAAAAAGATAATTACAGTTTTTGGGTCTATATACTACTCTAAAAATGTGGTGGTGGCTATTTTTTTGCTCGAATTCACTGACGGAGGGAAACCACTAAACTTCTCTATTGAAGAATT	Repair oligos
SLC1_dg fw	AGCGATGAGATGCGACTCTG	Diagnostic primers for seamless deletion confirmation
SLC1_dg rv	GTCGAGGAGGTTCTGCCATC	Diagnostic primers for seamless deletion confirmation

Table S4 Relative TAG content (<2%) of 7 fruits.

	TAG content (Area %)						
	T1	T2	T3	T4	T5	T6	T7
ALiO	1.5	1.1	1.4	0.7 ± 0.14	0.4 ± 0	0.35 ± 0.07	0.1 ± 0
POP	1.2	0.7	0.1	0.2 ± 0	0.55 ± 0.07	0.3 ± 0	0.2 ± 0
PLiP	0.8	1.2	1.6	0.2 ± 0	0.4 ± 0	0.2 ± 0	0.1 ± 0
SLiA	0.8	0.1	0.1	0.45 ± 0	0.3 ± 0.07	0.25 ± 0	0.3 ± 0
PPM	0.7	0.1	0.1	0.2 ± 0	0.15 ± 0.07	0.1 ± 0	0.1 ± 0
PPP	0.6	0.1	0.1	0.4 ± 0	0.4 ± 0	0.35 ± 0.07	0.2 ± 0
BehOO	0.6	0.1	0.1	0.3 ± 0	0.2 ± 0	0.2 ± 0	0.1 ± 0
PLeO	0.5	1.2	1.2	0.25 ± 0.07	0.3 ± 0	0.15 ± 0.07	0.1 ± 0
AOA	0.4	0.1	0.1	0.45 ± 0.21	0.1 ± 0	0.15 ± 0.07	0.2 ± 0
BehLiO	0.4	0.1	0.1	0.4 ± 0	0.4 ± 0.14	0.3 ± 0	0.1 ± 0
LigLiLi	0.4	0.8	0.1	0.15 ± 0.07	0.15 ± 0.07	0.1 ± 0	0.4 ± 0
PLeP	0.2	0.6	0.6	0.1 ± 0	0.1 ± 0	0.1 ± 0	0.1 ± 0
LiLiO	0.2	0.7	0.1	0.1 ± 0	0.1 ± 0	0.1 ± 0	0.1 ± 0
LeOO	0.2	0.7	0.4	0.1 ± 0	0.1 ± 0	0.1 ± 0	0.1 ± 0
MOP	0.1	0.1	0.1	0.1 ± 0	0.1 ± 0	0.1 ± 0	0.1 ± 0
MOO	0.1	0.1	0.1	0.1 ± 0	0.1 ± 0	0.1 ± 0	0.1 ± 0
PPS	0.1	0.1	0.1	0.1 ± 0	0.1 ± 0	0.1 ± 0	0.1 ± 0
PSS	0.1	0.1	0.1	0.1 ± 0	0.1 ± 0	0.1 ± 0	0.1 ± 0
SSS	0.1	0.1	0.1	0.15 ± 0.07	0.15 ± 0.07	0.2 ± 0	0.8 ± 0
LiLiLi	0.1	0.1	0.1	0.1 ± 0	0.1 ± 0	0.1 ± 0	0.1 ± 0
LigLiO	0.1	0.1	0.1	0.1 ± 0	0.1 ± 0	0.1 ± 0	0.1 ± 0

Table S5 TPM values of potential Shea tree TAG biosynthetic genes.

	Annotated probable gene	T3[a]	T6	Ratio[b]
VpGPAT1	Glycerol-3-phosphate acyltransferase 3	69.68	103.98	1.49
VpGPAT2	Glycerol-3-phosphate 2-O-acyltransferase 6	18.82	2.69	0.14
VpGPAT3	Probable glycerol-3-phosphate acyltransferase 8	8.38	0.77	0.09
VpGPAT4	Glycerol-3-phosphate acyltransferase	6.56	2.8	0.43
VpLPAT1	Probable 1-acyl-sn-glycerol-3-phosphate acyltransferase 5	23.54	8.92	0.38
VpLPAT2	1-Acyl-sn-glycerol-3-phosphate acyltransferase 2	19.03	10.99	0.58
VpLPAT3	Lysophospholipid acyltransferase LPEAT2	9.59	1.58	0.16
VpLPAT4	Lysophospholipid acyltransferase 1	13.9	0.95	0.07
VpLPAT5	1-Acyl-sn-glycerol-3-phosphate acyltransferase 1, chloroplastic	8.88	10.62	1.20
VpLPAT6	1-Acyl-sn-glycerol-3-phosphate acyltransferase 2	57	27.94	0.49
VpLPAT7	Lysophospholipid acyltransferase LPEAT1	28.63	13.86	0.48
VpLPAT8	Lysophospholipid acyltransferase LPEAT2	3.33	2.99	0.90
VpDGAT1	Phospholipid:diacylglycerol acyltransferase 1	14.84	3.86	0.26
VpDGAT2	Acyltransferase-like protein At1g54570, chloroplastic	4.29	0.09	0.02
VpDGAT3	Phospholipid:diacylglycerol acyltransferase 1	12.94	6.78	0.52
VpDGAT4	Acyltransferase-like protein At1g54570, chloroplastic	5.06	1.67	0.33
VpDGAT5	Diacylglycerol O-acyltransferase 1	14.45	16.3	1.13
VpDGAT6	Phospholipid--sterol O-acyltransferase	4	5.57	1.39
VpDGAT7	Diacylglycerol O-acyltransferase 2	9.32	7.08	0.76
VpDGAT8	Flap endonuclease 1	3.16	1.86	0.59
VpDGAT9	Acyltransferase-like protein At1g54570, chloroplastic	5.06	1.67	0.33
VpDGAT10	Lecithin-cholesterol acyltransferase-like 4	3.29	1.73	0.53
VpDGAT11	Phospholipase A(1) LCAT3	48.62	34.55	0.71

[a], TPM (Transcripts Per Kilobase Million) value. [b], TPM ratio of T6/T3.
